# Neurobiological Effects of Binge Drinking Help in Its Detection and Differential Diagnosis from Alcohol Dependence

**DOI:** 10.1155/2018/5623683

**Published:** 2018-07-04

**Authors:** Napoleon Waszkiewicz, Beata Galińska-Skok, Anastasiya Nestsiarovich, Agnieszka Kułak-Bejda, Karolina Wilczyńska, Katarzyna Simonienko, Mikołaj Kwiatkowski, Beata Konarzewska

**Affiliations:** ^1^Department of Psychiatry, Medical University of Białystok, Plac Brodowicza 1, 16-070 Choroszcz, Poland; ^2^Center for Global Health, Department of Internal Medicine, University of New Mexico Health Sciences Center, BRF No. 323H, MSC10-5550, 915 Camino de Salud NE, Albuquerque, NM 87131, USA

## Abstract

The prevalence of binge drinking in the general population is 3-4 times higher than that of alcohol dependence. Neuroimaging studies show that binge drinking in adolescence impairs brain development and white matter integrity. Regions with reduced functional activity include the limbic system, ventral diencephalon, frontal lobe, and middle and inferior temporal lobes, whereas the right superior frontal and parietal lobes are typically hyperactivated. The observed activation of the frontoparietal areas might reflect the alternative memory system operating, whereas the reduced occipito-hippocampal response is associated with impaired visual and linguistic processing/learning. Some other findings from literature research include a decrease of N-acetylaspartate (NAA) in the frontal lobe and its increase in the parietal lobes, as well as the reduced components of event-related potentials, reflecting deficit in attention, working memory, inhibition, and executive functioning. Animal studies show that even a single day of binge drinking results in a neurodegeneration and reactive gliosis in the limbic cortex as well as in gene expression dysregulation and histone acetylation. Another biological evidence on binge drinking effect include inflammatory response, oxidative stress, formation of toxic ceramides, activation of caspase 3, and secretion of corticoliberin. Some of the binge drinking-induced cognitive abnormalities can be reversible after three weeks of abstinence. Although binge drinkers have a similar pattern of neuropsychological deficits with chronic alcohol consumers (mainly memory deficits), binge drinkers have prominent impairment of inhibitory control, which may be a marker of binge pattern of alcohol drinking. The optimal therapeutic strategies should target the inhibitory control processes to facilitate discontinuation of alcohol consumption and to block its possible progression to the alcohol dependence syndrome.

## 1. Introduction: Definition and Epidemiology of Binge Drinking

Worldwide, 6.2% of mortality and 7.4% of morbidity among men and 1.1% of mortality and 1.4% of morbidity in women are associated with alcohol consumption. Alcohol abuse contributes to about 2.5 million of deaths annually and is the third risk factor for morbidity and disability (and is the first factor in middle-income countries) [1–9]. Binge drinking (also called a “risky single-occasion drinking,” “heavy sessional drinking,” or “heavy episodic drinking”) is the dominant pattern of alcohol consumption among young adults, typically practiced in the evenings and on weekends [10, 11]. Over the last several years, many definitions of binge drinking have appeared. The previous definition was characterizing it as heavy drinking during 2–7 days. New definition characterizes it as an episodic drinking of more than 5 units of alcohol for men and 4 units for women at once (1 unit = 10 g of pure ethanol) [11–17]. Given the fact that there are international differences in the definition of the “unit” or “standard drink,” based on the amount of pure ethanol required (8 g for UK, 10 g for Poland and Australia, 14 g for USA, and ~20 g for Japan) and that there are sex differences in alcohol metabolism and distribution of alcohol [3], a new comprehensive definition of “binge drinking” was proposed by the US National Institute on Alcohol Abuse and Alcoholism agency [18]: it is a pattern of drinking resulting in blood alcohol concentration (BAC) of 0.08 gram percent or above. This typically corresponds to consumption of 4 standard drinks or more for women and 5 drinks or more for men taken during 2 hours. The other binge drinking definitions include consumption of more than a half of the recommended weekly limits of alcohol intake (1–14 drinks for a female and 1–21 drinks for a male) or occasional consumption of more than double of the recommended maximum daily dose of alcohol (the recommended maximum daily dose is 1-2 units for females and 2-3 units for males) [12, 19].

According to the WHO data, the problem of binge drinking affects more than 7% of the world population, more than 13% in America, and more than 16% in Europe, being the dominant pattern of alcohol consumption among adults. Every third person consuming alcohol is a binge drinker [2]. More than 2% of the world population is alcohol dependent, 3.4% in America, and 4% in Europe. Therefore, it is suggested that the prevalence of binge drinking is 3-4-fold higher as compared to alcohol dependence [2, 6, 15].

The peak prevalence of binge drinking occurs in late adolescence and young adulthood [20]. The evidence shows that the earlier the onset of binge drinking, the greater the risk of subsequent alcohol use and its associated comorbidities in adulthood [21, 22]. Although young people typically consume alcohol less frequently than older people, they tend to drink larger amounts at one occasion [23]. A greater risk of binge drinking was shown for Caucasians who have never been married, placed little importance on religion, or had their grade point average of B or less [24]. African Americans and Asians are less prone to practice binge drinking, which can be explained by the inactive form of the enzyme acetaldehyde dehydrogenase (ALDH2), resulting in the rapid accumulation of a toxic substance—acetaldehyde, “flushing response,” and subsequent “next-day effect”—“hangover” [25–27].

Among the individuals who frequently practice binge drinking, there is a 13-fold or 19-fold increase in the risk of being classified as an alcohol-abusing or alcohol-dependent person, respectively. Among young binge drinkers who have more than 3 binge drinking sessions in a week, the risk of alcohol dependence is from 2 to 5 times higher as compared to the general population [20, 21]. More than 80% of episodes of binge drinking are observed in males, and males are more often dependent on alcohol than females [25]. However, some researchers still believe that binge drinking among young people is a poor predictor of the development of alcohol-related disorders in the future, as those individuals describe their excessive alcohol consumption as rather symptomatic [20].

Binge drinking contributes also to injuries (falls, traffic accidents), alcohol poisoning, myocardial infarction, atrial fibrillation, stroke, sexually transmitted diseases, birth defects, violence (including domestic), unintended pregnancies, suicide, drowning, sudden infant death syndrome, and so on [10–19].

## 2. Review: The Harmful Effect of Binge Drinking on the Brain

Analysis of the published research literature showed that the following features are associated with current binge drinking: disruptive family events, personality trait of agreeableness, impulsivity, pubertal status, and a tendency to devalue future rewards [28]. The factors associated with both current and future binge drinking include romantic and sexual relationship life events (which also might result from getting drunk), novelty seeking, disorderliness, extravagance, and conscientiousness. According to the latest research, biological predictors of binge drinking include changes in brain parenchymal volume and the grey/white matter ratio and, in particular, changes in the right middle and precentral gyri and in the bilateral superior frontal gyrus [29, 30]. Among the predictors of binge drinking in adolescents are reduced grey matter volume and increased reward-related activity in superior frontal gyrus and increased volume and activity in the premotor cortex, when failing to inhibit the response. The most robust endophenotypes associated with binge drinking are reduced volume of the ventromedial prefrontal cortex (vmPFC), its decreased activity when anticipating or receiving reward or increased when processing angry faces, and reduced volume and activity of the left inferior frontal gyrus (IFG) when anticipating reward and when processing angry faces. Therefore, binge drinking is accompanied with abnormalities in brain parts of emotional and behavioral regulation [28]. It was demonstrated that even 1-2 alcohol intoxication(s) by age 14 is sufficient to predict the subsequent development of binge drinking, and each year delay in the onset of alcohol consumption in adolescents reduces the risk of the alcohol dependence in the adulthood by 10%.

Among the etiological factors of binge drinking in young adults is the genetic predisposition to alcohol abuse/dependence and the accompanying reduced sensitivity to the effects of alcohol intoxication (a higher tolerance) [31]. Therefore, individuals homozygous for the ALDH2 gene appear to be protected against binge drinking due to high concentrations of acetaldehyde and the subsequent hangover effects. It was shown college students with the short variant of the serotonin transporter gene (5-HTT) consume more alcohol during one occasion and get intoxicated more frequently compared to students with the normal gene variant. It is often interpreted as a “self-medication” behavior targeting anxiety and depression in binge drinkers. Other gene candidates that might contribute to binge drinking behavior include ras-specific guanine nucleotide-releasing factor 2 (RASGRF2), Snapc3, EHD4, or EDH1. Most of these genes control mesolimbic dopamine functioning in the ventral tegmental area (VTA) that sends projections to the nucleus accumbens (NAC) [32]. G protein-coupled inwardly rectifying potassium (GIRK) channels are the critical regulators of the neuronal excitability and can be directly activated by the ethanol, and its GIRK3 expression in the VTA is modulated by binge drinking [33].

The reduction in components of the event-related potentials (ERP) was observed in binge drinkers. ERP are electroencephalogram (EEG) changes that have very small voltages generated in the brain structures in response to the specific events or stimulation. N1, MMN, and especially the P300 component can reflect impairments in attention, working memory, response inhibition, and executive functioning (e.g., making decisions) [31, 34, 35]. A higher EEG density of the beta and theta oscillations found in the right temporal lobe and bilateral occipital cortex of binge drinkers may potentially lead to difficulties in cognitive processing and decreased response to stimulation, which in its turn may result in an inability to proper understanding of the information coming their way, for example, when to stop drinking [36].

Despite the evidence of brain structural and functioning abnormalities associated with binge drinking, it is not always clear whether these endophenotypes derived from neuroimaging, biochemical, neurophysiological, and neuropsychological studies are the cause or the effect of binge drinking.

The catalase is the primary enzyme that metabolizes ethanol to acetaldehyde in the brain [37]. The central nervous system dysfunction in binge drinking results from a direct action of ethanol and its metabolites (acetaldehyde, reactive oxygen species (ROS)) or congeners (methanol) on brain metabolism, electrical properties of the membranes, the number of microglial cells (activation of microglia and immune response in the cerebellum), and immunogenicity of lipopolysaccharide (LPS) [7, 38]. Binge drinking was shown to affect neurotransmission of glutamine (related to cytotoxicity in the nucleus accumbens), dopamine (also mediated by endocannabinoid mechanisms), the opioid system (increased apoptosis of neurons containing *β*-endorphin in the arcuate nucleus of the hypothalamus), and acetylcholine, affects proteins that regulate formation of the neurotransmitters (nuclear factor (NF) KB (NFKB), cyclic AMP-responsive element-binding protein (CREB)), and is likely to be involved in the serotonin and gamma-amino-butyric acid (GABA) pathway abnormalities [38, 39]. Such disturbed neurotransmission may result from the abnormal protein synthesis, given that a selective reduction in brain-protein synthesis was observed in the cortex, cerebellum, and the brain stem [39]. A spared metabolism of the midbrain might be due to a better stability of antioxidants such as glutathione or superoxide dismutases [40]. Even a single day of alcohol intoxication in rats increases the piriform cortex neuron degeneration, which functionally connects the olfactory and orbitofrontal cortexes. It may explain the lack of inhibition and thus continuation of alcohol drinking by binge drinkers [41, 42]. There was also observed reactive gliosis in the hippocampus. The astrocytes appear to be more susceptible to toxic effects of ethanol than neurons, and it is speculated that they are responsible for the subsequent deterioration of neurons [41].

Common next day effects (hangover) include fatigue, weakness, thirst, headache and muscle aches, nausea, abdominal pain, sleep disorders (reduction of rapid eye movement phase), dizziness, sensitivity to light and sound, attention and concentration disorders, decreased mood, anxiety, irritability, tremor, sweating, increased blood pressure and heart rate, and aversion to alcohol. These hangover symptoms may be explained by (a) the direct effects of alcohol—dehydration (inhibition of antidiuretic hormone), electrolyte imbalance, gastrointestinal disturbances, hypoglycemia, sleep, and biological rhythm disturbances; (b) increased levels of acetaldehyde and ROS; and (c) other factors such as alcohol metabolites/congeners (methanol), concomitant use of other substances (e.g., nicotine), personality disorders, history of alcohol addiction in the family, and glutamine or vitamin B12 deficiency [43].

Although it was found a transient neuroprotective effect of binge drinking (conversion of ceramides to sphingomyelin), the deleterious binge drinking-effects on the brain dominated. These "bad" effects include inflammatory response, oxidative stress (mainly in astrocytes), formation of toxic ceramides (remodeling), activation of caspase 3, and functional disconnection of the medial prefrontal cortex and the central nucleus of the amygdala [44]. The other abnormalities include the dysfunction of the GABAergic system of neurotransmission, impaired secretion of the corticoliberin, and impaired executive functioning control over motivated behavior. Alcohol can be metabolized into acetaldehyde locally in the hippocampus, causing the synthesis of neurosteroids and changes in neuroplasticity [45]. Glutamatergic neurons can increase their own GABAergic inhibitory responses in a paracrine or autocrine manner under significant acute stimulation or stress. Therefore, the memory blackout characteristic for acute alcohol intoxication seems to reflect a homeostatic neuroprotective adjustment of some of the glutamatergic neurons [45]. It was also found that binge drinking may potentiate glutamatergic neurotransmission, and its subsequent shrinkage may cause alterations in synapse number and dendritic spine morphology during adolescence and young adulthood, which can make the neurons vulnerable to future excitotoxic activation. Alcohol poisoning can also result in degeneration of neurons, delayed maturation of the GABAergic system, and decreased GABA receptor density in the dorsal striatum [46–48]. In rats exposed to 4-day repeated binge-like alcohol cycles, the transient enlargement of the cerebrospinal fluid volumes of the lateral ventricles and cisterns was accompanied by the transient decrease in N-acetylaspartate (NAA) (marker of cell vitality) and total creatine (index of osmotic balance/energy utilization) and the increase in the choline- and glutamate-glutamine-containing compounds. Such pattern of alcohol exposure also resulted in the neuron loss in the olfactory, entorhinal, perirhinal, and piriform cortexes and the increased amount of microglia [49]. It has also been demonstrated that even a single binge-like exposure to ethanol in rodents (3 g of ethanol/kg) was associated with up- and downregulation of genes in the cerebral cortex, nucleus accumbens, and ventral tegmental area, increased histone acetylation in the amygdala; and altered expression of genes that modify histones in the striatum and prefrontal cortex, leading to chromatin remodeling in the hippocampus [50].

Repeated sessions of binge drinking result in cognitive, behavioral, and biochemical defects and in the neurodegeneration in animals. However, the extrapolation of these findings to humans must be done with caution. The rats have a shorter life cycle than humans and one-day rat feeding with alcohol may correspond to a longer period of alcohol consumption in humans. The administration of alcohol to the rats is often forced and, thus, stressful; moreover, the doses of alcohol used in animal experiments are several folds higher (7-13 g/kg) than those used in humans (0.7 g/−2 g/kg) [12, 22, 41]. In addition, deteriorating effects of alcohol on animals and humans are mediated by the duration of ethanol exposure, dosage, and brain region [7].

## 3. Binge Drinking and Brain Development

The maturation of the brain is associated with transition from intense and diffuse neural activity to a less intense and more focal cortical activation (neural efficiency). It is accompanied by reduction in synaptic density along with myelination processes, which improves cognitive abilities including working memory and inhibitory control [51]. In general, young people drink less often than older adults but they drink greater amounts of alcohol per occasion. The highest rate of binge drinking occurs at the age of 21–24 years, exposing individuals to an acute damage to their health [23, 31]. Adolescents and young adults are more sensitive to alcohol effects, which results in the asynchronous development of the prefrontal cortex with respect to the limbic system [23]. The neuroimaging studies found that the earlier maturation of the reward area (NAC) as compared to the cognitive control cortex (PFC) contributes to decision making—to consume alcohol. Adolescents also have higher dopamine release in the striatum than adults, which appears to be associated with greater rewarding effects of binge drinking. Young people are also more likely to die because of the acute effects of alcohol than its chronic effects. Therefore, the abstinence in adolescence (13–18 years) and young adulthood (19–24 years) is of particularly high importance for their health, as the temporal and prefrontal cortexes are the cortical regions where the full maturation of the grey matter appears in the last turn (until the age of 20–25 years) [47]. The reduced cortical thickness was reported in pruning (synapse elimination/maturation) regions of the cortex in young adults, for example, in the superior frontal and temporal gyri, as well as in the regions that have finalized their maturation (precentral and supramarginal gyri). The increased risk of neurocognitive dysfunction coupled with the hippocampus and entorhinal cortex degeneration was found in subjects with frequently repeated episodes of binge drinking [47]. It has been shown that young people are more vulnerable to the alcohol-induced impairments in memory, attention, cognitive processing, and language skills than older ones [52].

## 4. Neuroimaging, Neurocognitive, and Neurophysiological Studies and Binge Drinking

The magnetic resonance imaging (MRI) studies showed that binge drinking in young adults (18–24 years) is associated with the reduced frontal thickness of the right middle anterior cingulate cortex (mid-ACC) and the left dorsal posterior cingulate cortex (dPCC). Binge drinking episodes due to their acute neurotoxic effect might interfere with brain maturation, increasing the microarchitectural prunning and the subsequent loss of neurons [47]. The damage of binge drinking to specific brain regions results in the related functional abnormalities: for example, the thinning of the right mid-ACC might result in impairments of the response selection, assessment of risk, cognitive control in decision-making, reward anticipation, error detection, and conflict monitoring. The thinning of dPCC might result in impairments in behavioral adaptation to changes of the environment, control over the attentional focus and vigilance, appraisal value of the reward and its consequences (e.g., alcohol), and coordination of the other brain regions involved into task solving. The thinning of the two of the abovementioned brain regions was significantly associated with the amount of the alcohol consumed during a single drinking session and with the total amount of alcohol consumed [47]. Squeglia and colleagues [53] found that binge drinking in adolescents can lead to a smaller left cingulate gyrus, pars triangularis, and rostral anterior cingulate volume in adulthood, as well as a reduced right cerebellar white matter volume. During 3 years of follow-up, the individuals practicing heavy drinking have reduced volumes in the left ventral diencephalon, left inferior and middle temporal gyri, left caudate nucleus, and brain stem, when compared to the substance-naive youth. These findings are consistent with the results of the earlier studies revealing the preexisting cognitive deficits in tasks involving frontal regions and the downstream consequences of binge-drinking on brain regions involved in language and spatial tasks. It was also shown that even normative alcohol use in adolescence is associated with brain abnormalities: reduced volume of the amygdala, increased volume of the cerebellum, and reduced cortical volume and thickness of the frontal and temporal regions (superior and middle frontal gyri, pars triangularis, middle and inferior temporal gyri, and right hippocampus) [54, 55]. Sousa and colleagues showed that higher grey matter densities in the left middle frontal gyrus in binge drinkers were associated with impulsiveness scale [56]. The data suggest that there exist certain vulnerability factors predisposing an individual toward the initiation of heavy alcohol use, impulsiveness, and drinking continuation in binge drinkers [46–48].

A diffusion tensor imaging (DTI) is a noninvasive MRI which allows visualizing and evaluating the integrity of the white matter by a quantitative and directional analysis of free diffusion of water molecules in the extracellular space of the tissue. Fractional anisotropy ratio (FA), which is a marker of the white matter integrity, was shown to be reduced in the frontal, temporal, and parietal lobes; cerebellum; corpus callosum; internal and external capsules; superior longitudinal fasciculus; corona radiata; commisural limbic; brainstem; and cortical projection fibers in young binge drinkers. The reduced white matter integrity in the prefrontal, parietal, occipital, and temporal segments of the corpus callosum were generally negatively correlated with the incidence of binge drinking, and males were more susceptible to the negative alcohol effects than females due to the later white matter maturation [23, 48, 57–59]. The reduced axial diffusivity, found in rodents' neocortex, hippocampus, and cerebellum, is interpreted as the indication of the axonal injury and/or reduced axonal density which can lead to subsequent dysregulation of the prunning process, myelination, cognitive dysfunction, and anxiety-like behavior. The observed radial and mean diffusivity reduction can result from the extracellular matrix reorganization with increased tissue density [48]. Some studies have also reported a binge-drinking-associated increase in brain connectivity and FA of the cingulum (the area which is often associated with reward and positive reinforcement), in the corona radiata and capsula [60–62], or increase in the neurite density in the cortical white matter [63]. Such observed brain hyperconnectivity might place these individuals at higher risk of impulsive behavior, including binge drinking. An observed reduced orientation dispersation index (ODI) in the frontal cortical grey matter and a greater ODI in the parietal and ventral striatal grey matters in binge drinkers may point to changes in neurite density in these structures [63].

Functional magnetic resonance imaging (fMRI) studies have shown that binge drinking is associated with changes in activation (assassed by the blood-oxygen-level-dependent signal (BOLD)) in the resting brain including the subcallosal cortex (SCC), left temporal fusiform cortex (TFC), and left inferior temporal gyrus (ITG), which is involved in the reward brain network, visual recognition of the emotions, and memory, respectively [64]. The greater activation in the right superior frontal and bilateral posterior parietal cortexes or reduced activation in the dorsolateral, dorsomedial prefrontal, anterior cingulate, and occipito-hippocampal cortexes was observed in other studies [65, 66]. It was suggested that the reduced occipito-hippocampal response in binge drinkers reflected the lack of the visual and linguistic processing when learning verbal material, whereas a greater fronto-parietal response reflected the activation of the alternative memory system. There was also observed increased activity of the left amygdala and insula bilaterally while making decisions, indicating the dysfunction/hyperreactivity of the neural system implicated in the execution of emotional and incentive-related behaviors [67]. The increase in the neural activity related to verbal learning, decision making, working memory, and inhibitory control in young binge drinkers is also interpreted as a compensatory brain activation, which allows these individuals to remain behaviorally asymptomatic [51]. The fMRI findings also revealed the alterations in the cerebellum and the decreased activation of dorsal caudate nucleus during the risky decision making [46].

The magnetic resonance spectroscopy (MRS) studies of binge drinking have shown an associated decreased concentrations of NAA and the increased metabolism and loss of white matter in the frontal lobes, as well as greater NAA concentrations in the parietal grey matter. The alcohol consumption correlated with the impaired executive functioning and working memory, which in its turn correlated with the reduced frontal NAA signal [68]. This may be indicative of the neuronal loss in the frontal lobes and the relatively less damage to parietal neurons in binge drinking individuals [31].

The neurophysiological studies have also revealed the brain dysfunctions associated with binge drinking. Electroencephalography (EEG) studies found the alterations of the delta and fast-beta activity in binge drinkers, which resembles the EEG spectral pattern in alcohol-dependent individuals. The relative observed increase in the fast beta-rhythm may be a biomarker for future alcohol dependence studies [69]. The increase in beta (right temporal cortex) and theta (bilateral occipital cortex) oscillations observed in young binge-drinking individuals can indicate the increased cortical excitability and potential difficulties in the information processing capacity [36]. The event-related potentials (ERPs) measured with electroencephalography reflect the brain's direct response to a specific sensory, cognitive, or motor event. Anomalies of the amplitude and/or latency values of ERP components P1/N1, N2/P3, P3, and NoGo-P3, which reflect perception, attention, working memory, and inhibitory control, respectively, were described in binge drinkers. ERPs revealed a decrease in the P3a component latency during a facial discrimination task. Binge drinkers engaged in visual working memory tasks had also anomalies in the N2, P3, and the late positive (LP) components and underactivated right anterior prefrontal cortex [51, 58].

The neuropsychological studies seem to confirm the data from neuroimaging and neurophysiological studies in binge drinkers, in particular, the dysfunction of the frontal lobe such as the spatial working memory and the pattern recognition deficit [31, 70]. The deficit of the frontal inhibitory control is often observed in binge drinkers (more profound in females than in males), as well as the executive functioning deficit (planning). The Iowa Gambling Task (IGT) revealed significantly more mistakes in decision making associated with the dysfunction of vmPFC in binge drinkers, as well as the overactivation of the insula bilaterally [46]. The deficit in episodic and verbal memory was observed in binge drinkers, as well as the impairments in the visual memory, vigilance, and sustained attention which are related to temporal lobe dysfunction. Moreover, women had worse cognitive plasticity than men, which could result from the less-effective alcohol metabolism [31].

The studies on hangover effects of alcohol on memory found that the processes of encoding and consolidation are disturbed in the studied individuals whereas the delayed recall ability is intact. It was also suggested that the retrieval is disturbed only during hangover. This prolonged effect of the alcohol hangover state on human's cognitive functioning is well described in the US Federal Aviation Administration's Pilot Safety Guidelines that includes the statement “eight hours from bottle to throttle” (throttle means the desired power level of the airplane's engine). There is also evidence that young binge drinkers have persistent memory deficits even three weeks after their last drink [31]. Binge drinkers performed significantly worse on the executive functioning tasks as compared to nondrinking individuals in both young and elderly age groups, making significantly more perseverative errors which were shown to be related to the intensity of alcohol use [23]. Although binge drinking is associated with the impairments in decision making, no association is observed with age of alcohol consumption onset and impulsivity. However, the cognitive deterioration characteristic for binge drinkers may be partially attributed due to the increased levels of anxiety and depression.

## 5. Additional Proofs: Prospective Studies and the Associations Revealed with the Amount of Alcohol Consumed

Just a few prospective studies have focused on binge drinkers who had no previous history of alcohol consumption. During the 3 years follow-up, the following were observed in binge drinking adolescents: reduced visuospatial memory, sustained attention, and impaired activation of the frontal, parietal, and occipital lobes during tasks involving visual working memory [71, 72]. In another prospective electrophysiological study, the delayed latencies were found for P100, N200, and P300 components of the ERPs in binge drinkers [73]. Such prospective studies clearly demonstrate a real brain dysfunction in the absence of any preexisting organic brain damage. When binge drinkers who consumed 15–59 portions of alcohol in 2-3 sessions per week were compared with individuals with the same weekly alcohol intake but spread to a daily drinking (5–7 occasions), it was found that the first group had more profound neurophysiological and cognitive dysfunction than the second one [74].

The association was found between the earlier onset of binge drinking and the poorer decision making. The association was also found between amounts of alcohol and the smaller cerebellar volume and the higher activity of dorsomedial prefrontal cortex [51, 58]. Also, an association was found between the earlier onset of regular drinking and the greater quantity and intensity of alcohol consumption and the larger P3b amplitude of the evoked potentials. Thus, attention and working memory, described in the literature, seem to be more vulnerable to the effects of chronic heavy alcohol drinking, while binge drinking seems to impair the inhibitory control at most [51].

## 6. Binge Drinking versus Chronic Drinking

The computed tomography and MRI revealed the increased brain ventricles and reduced volume of the frontal and prefrontal cortexes and hippocampus in alcohol-dependent individuals. DTI studies found increased FA values and the decreased mean diffusivity in the corpus callosum, as well as a negative correlation between the number of years of alcohol consumption and FA in the internal capsule, superior longitudinal fasciculus, and other white matter tracts [59]. The fMRI studies showed the increased BOLD signal in the prefrontal area (mostly in females), the MRS studies showed the increased glutamate levels in the anterior cingulate cortex and decreased NAA levels in the frontal lobe and cerebellum of alcohol-dependent subjects, which have normalized after 5-6 weeks of abstinence with an accompanying improvement in verbal learning, memory, and attention. The increased fluid volume associated with chronic drinking is due to the so-called brain shrinking phenomenon, which is reversible along with alcohol abstinence [23, 75]. The cognitive impairments found in alcohol-dependent subjects include visual-spatial deficits, executive disfunction (problem solving, mental flexibility, and response inhibition), impared attention and working memory, and impairment of “fluid cognitive abilities” (e.g., concept formation). These defects might result from the kindling mechanism, which is associated with disturbed function of the GABA system and hyperactivity of the glutamatergic system. It was found that spontaneous recovery of cognitive functions in alcohol-addicted individuals occurs within the first six months of abstinence [23, 76].

Some studies suggest that the substantial cognitive deficits occur after 10 years of the sustained alcohol use [77]. It is also assumed that brain impairments in binge-drinking subjects are consistent with those seen in alcohol-dependent individuals, for example, changes in the volume of the cerebellum, thickness of the cerebral cortex, integrity of the white matter, EEG and evoked potentials (component P3), and neurocognitive deficits (brain activation during verbal learning, working memory, and decision making) [23]. The similar pattern of cognitive deficits in binge and chronic alcohol drinkers includes mainly the impaired memory. It was even concluded that deficits in executive planning and episodic memory in binge drinkers are similar to frontal deficits observed in patients with Korsakoff syndrome. It confirms the theory that consumption of alcohol, even in the form of binge drinking, contributes to brain aging, at least cognitively [23, 77].

Despite the similar brain dysfunctions found in both binge drinkers and alcohol addicts, the deficits specific for binge drinking were found in neuroimaging, biochemical, neurophysiological, and neuropsychological studies [58]. It demonstrates the need for further studies to better assess the risks associated with binge drinking. Similar patterns of brain dysfunctions in binge drinkers and alcohol addicts point to a nosological continuum between these two patterns and even resemble two stages of the same disorder. It is consistent with the current Diagnostic and Statistical Manual of Mental Disorders (DSM-V) and confirms the rationale of shifting from diagnostic categories of alcohol abuse and dependence to the term “alcohol use disorder” with mild, moderate, and severe severity, according to the number of observed symptoms [78, 79]. Thus, binge drinking seems to be not only an adolescence-related normative feature that declines with the increased responsibility in life such as employment, marriage, and parenthood, as some authors suggested [58]. It is also necessary to take into consideration that the neuroimaging, biochemical, neurophysiological, and cognitive impairments found in alcohol addicts' brain may result not only from the clear effects of alcohol but also from other factors associated with the prolonged alcohol consumption such as trauma and vascular changes or from other alcohol-associated somatic diseases (e.g., liver damage), age, or nutritional deficiencies (e.g., vitamins) [80].

## 7. Conclusions

There is no consensus in discrepancies about defining binge drinking in the scientific literature: first of all, there are different amounts of standard drink in binge drinking patterns (>5, >6, or >8) as well as different amounts of pure ethanol in a standard drink (8–20 g); second, there are differences in duration of the binge drinking sessions (from 2 to 6 hours, the whole day, or even two to four days) and drinking with or without a meal; third, there are differences in alcohol metabolism and its distribution rate depending on sex, weight, and age [20]. These variations indicate that it might be more convenient to accept the internationally standardized definition of binge drinking, for example, determined by BAC, rather than by the number of units of alcohol in a session. This corresponds to a definition developed by NIAAA [18].

There are convincing evidence that even a single session of binge drinking leads to reversible changes in the brain, detectable in imaging, physiological, biochemical, and neuropsychological studies. The main regions impaired by binge drinking are the limbic system, diencephalon, frontal, and middle and inferior temporal lobes. The right superior frontal and parietal lobes are shown to be hyperactivated. Although the direct association between binge drinking and cognitive impairment is not easy to demonstrate, the imaging studies give clear evidence that binge drinking in adolescence may impair brain development, especially the integrity of the white matter [23]. The animal studies have shown that even a single day of binge drinking leads to neurodegeneration in the limbic cortex (associated with learning and spatial memory) including the olfactory bulb, piriform, entorhinal and perirhinal cortexes, and dentate gyrus of the hippocampus [41]. Some of the binge drinking-induced cognitive abnormalities were restored during 3 weeks of the abstinence [51]. Therefore, it seems reasonable that more researchers should focus their attention on the binge drinking problem to improve the existing prevention strategies and target the neuromaturation process in adolescents and young adults. The optimal therapeutic programs should strengthen the inhibitory control processes to facilitate the discontinuation of alcohol consumption, given that repeated binge drinking sessions can induce the disinhibition and permanent changes, which can be shown in physiological, biochemical functioning and the histological structure of the brain, and can induce the progression to alcohol dependence [51]. Disordered inhibitory control may be a marker of binge pattern of alcohol drinking. The proven effectiveness of brief interventions in reducing alcohol abuse [10–15] can potentially reduce the prevalence of the phenomenon of binge drinking.
